# Supplementary material for: Lack of Oxygen and/or Glucose Differentially Potentiates Aβ40e22q- and Aβ42-Induced Cerebral Endothelial Cell Death, Barrier Dysfunction and Angiogenesis Impairment
Source: Cells. 2026 Feb 27;15(5):424. doi: 10.3390/cells15050424 (PMC12984660; doi:10.3390/cells15050424)
Supplement: Supplementary file 1 [file cells-15-00424-s001.zip › cells-4110038-supplementary.pdf]

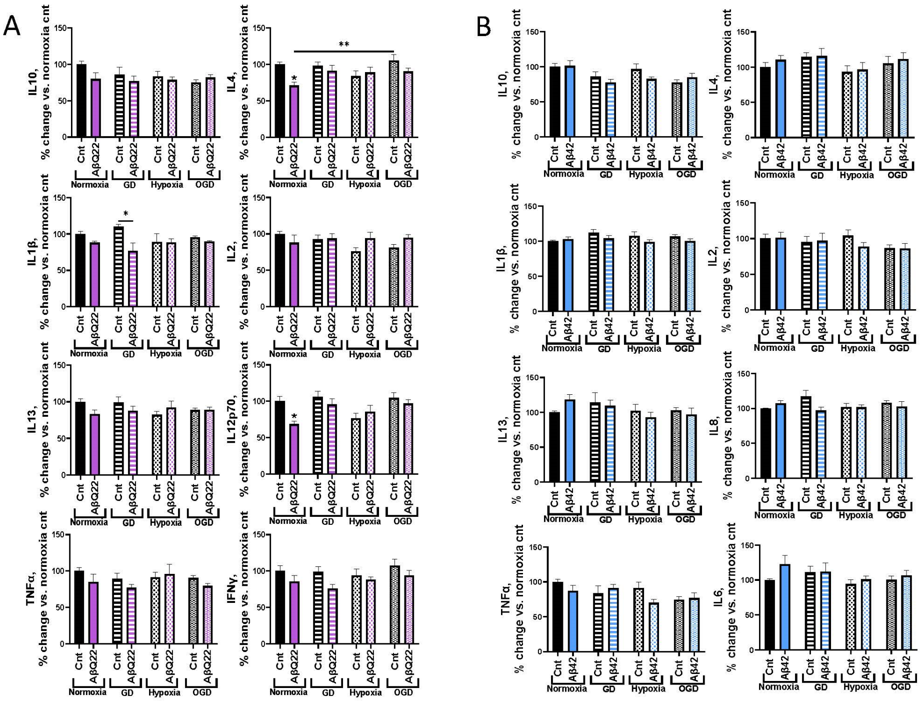

**Supplemental Figure S1. Modulation of BBB-related protein expression in HCMECs following acute treatment with AβQ22, Aβ42, and OGD. (A/B)** HCMECs were treated with 25μM AβQ22 **(A)** or 5μM Aβ42 **(B)**, GD, or a combination of both under conditions of normoxia or hypoxia for 6h. ZO1, ICAM1, pClaudin5, and MMP2 protein expression was evaluated via WB and actin was used for normalization. Data is represented as % change vs. normoxia control. (N=3 experiments with 2 technical replicates. Two way ANOVA, Tukey's post-test). *s located over bars are comparisons vs. normoxia control (****p<0.0001, ***p<0.001, **p<0.01, *p<0.05).

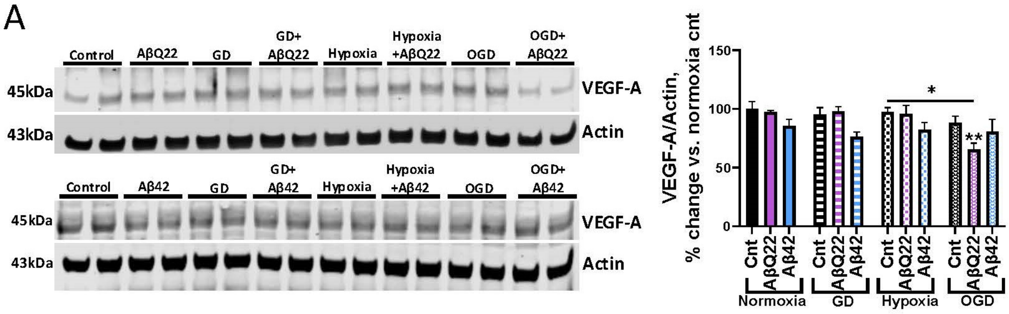


**Supplemental Figure S2. Effects of AβQ22, Aβ42, and OGD treatment on HCMEC release of additional pro-inflammatory cytokines.** (A/B) HCMECs were treated with 25μM AβQ22 (A) or 5μM Aβ42 (B), GD, or a combination of both under conditions of normoxia or hypoxia for 6h. Media was collected and utilized to run a multiplex pro-inflammatory cytokine assay (MSD). Protein concentration was utilized for sample normalization. Data is represented as % change vs. normoxia control (N=3 experiments with 2 technical replicates; one-way ANOVA, Tukey's post-test). *s located over bars are comparisons vs. normoxia control. (**** p<0.0001, ***p<0.001, **p<0.01, *p<0.05).

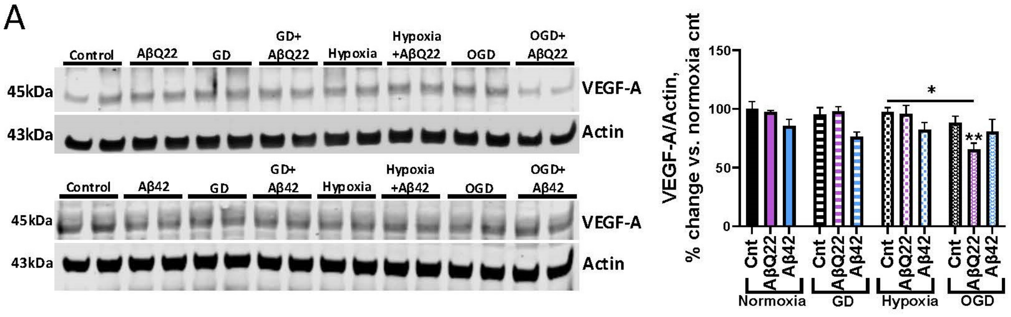

**Supplemental Figure S3. VEGF-A expressional changes following treatment with AβQ22, Aβ42, and OGD. (A)** HCMECs were treated with 25µM AβQ22 or 5µM Aβ42, GD, or a combination of both under conditions of normoxia or hypoxia for 6h. VEGF-A protein expression was evaluated via WB analysis and actin was used for normalization. Data is represented as % change vs. normoxia control. N=3 experiments with 2 technical replicates; two-way ANOVA, Tukey's post-test. *s over bars are comparisons vs. normoxia control (**** p<0.0001, *** p,0.001, ** p<0.01, * p<0.05).

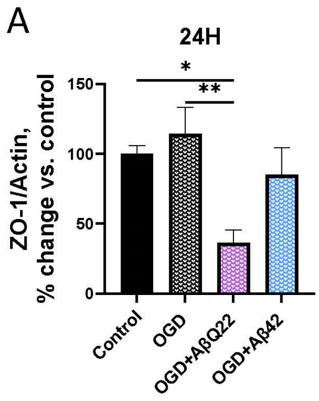


**Supplemental Figure S4. HCMECs treated with AβQ22+OGD reveal significantly decreased ZO1 protein expression compared to untreated controls as well as cells exposed to OGD alone. (A)** 24h ZO1 protein expression data from Figure 3 comparing only OGD treatment groups to untreated control cells. Data is represented as % change vs. normoxia control (N=3 experiments with 2 technical replicates; one-way ANOVA, Tukey's post-test). (**** p<0.0001, *** p<0.001, ** p<0.01, * p<0.05.

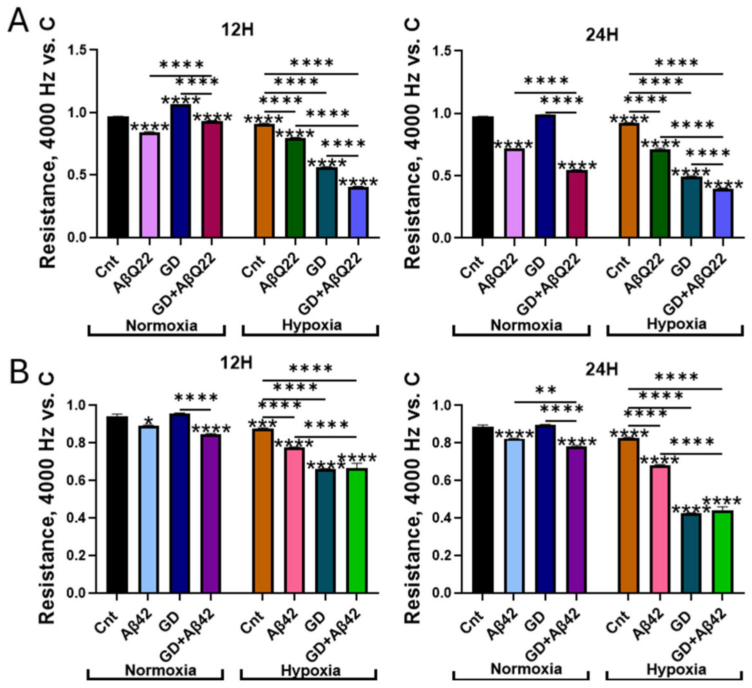


**Supplemental Figure S5. HCMEC ECIS TEER measurements represented as bar graphs at 12h and 24h. (A/B)** ECIS TEER data from Figure 2 represented as bar graphs for the 12h and 24h timepoints. TEER measurements recorded at each respective timepoint were plotted. (N=3 experiments with 2 technical replicates; two-way ANOVA, Tukey's post-test). *s over bars are comparisons vs. normoxia controls. (**** p<0.0001, *** p<0.001, ** p<0.01, * p<0.05).

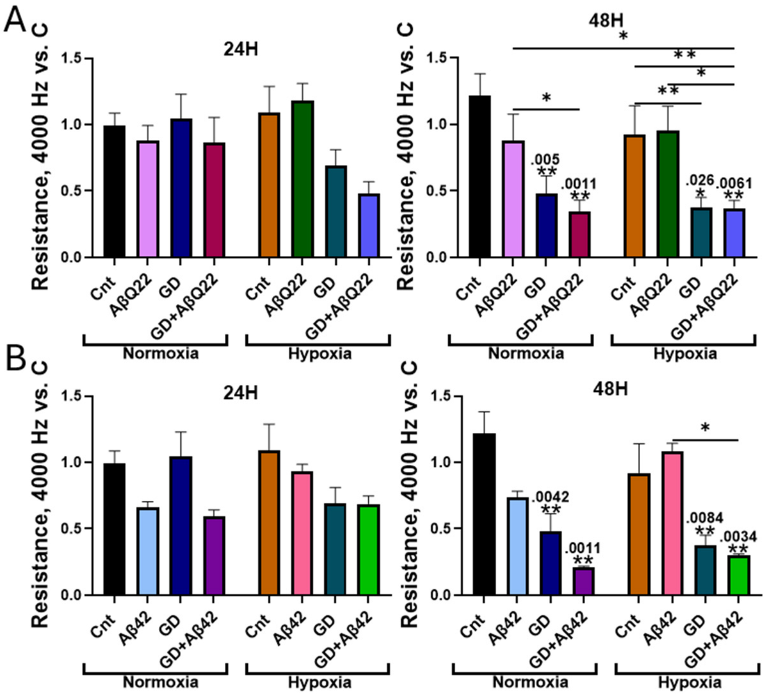


**Supplemental Figure S6. HCMEC ECIS wound healing measurements represented as bar graphs at 24h and 48h. (A/B)** ECIS wound healing data from Figure 2 represented as bar graphs for the 24h and 48h timepoints. Post-wound resistance measurements recorded at each respective timepoint were plotted. (N=3 experiments with 2 technical replicates; two-way ANOVA, Tukey's post-test). *s over bars are comparisons vs. normoxia controls. (**** $p<0.0001$, *** $p<0.001$, ** $p<0.01$, * $p<0.05$).
